# Development and Evaluation of a University-Based External Coaching Program for School Behavioral Health

**DOI:** 10.3390/bs16081391

**Published:** 2026-08-13

**Authors:** Katherine A. Perkins, Kristen Figas, Abigail Westbrook, Susan Barrett, Kimberly Yanek, Mark D. Weist

**Affiliations:** Department of Psychology, McCausland College of Arts and Sciences, University of South Carolina, Columbia, SC 29208, USA; kfigas@email.sc.edu (K.F.); westbra@email.sc.edu (A.W.); sb132@mailbox.sc.edu (S.B.); kyanek@mailbox.sc.edu (K.Y.); weist@mailbox.sc.edu (M.D.W.)

**Keywords:** systems coaching, school behavioral health, implementation support, interconnected systems framework, MTSS

## Abstract

School behavioral health (SBH) systems coaching is a developing approach to implementation support for the delivery of high-quality behavioral health support in schools through a multi-tiered prevention and intervention framework. Articulating coaching components and understanding how, when, and why external coaching supports systems change and implementation are essential, but research on the nature, scope, mechanisms, and effectiveness of external coaching for school behavioral health is limited. In the current study, we describe the development and implementation of the School Behavioral Health Academy (SBHA) Coaching program in South Carolina and present findings from a qualitative formative evaluation study. Thematic analysis of key informant interviews with district and school program implementation leaders (*N* = 8) provides evidence of perceived improvements in systems practices and positive staff and student outcomes related to participation in SBHA Coaching. Articulated barriers and facilitators point to potential mechanisms of change and implementation determinants. Participants described perceived changes and needs related to SBH understanding, infrastructure, norms, and mindsets, as well as the coaching skills and methods they found influential. Results are interpreted in relation to behavior change theory, systems change theory, and the Implementation Drivers Framework. Future directions for program development, research, and practice are discussed.

## 1. Introduction

In 2022, the South Carolina Department of Health and Human Services (SC DHHS) made policy changes to address a shortage of mental health counselors in schools, at the time an average counselor-to-student ratio of 1:1300 (South Carolina Department of Health and Human Services, 2026). Changes to Medicaid reimbursement schedules newly incentivized direct hire and private provider contracts for school behavioral health in addition to existing public mental health contracts, with higher rates offered to licensed clinicians than unlicensed. The state average mental health counselor ratio has notably improved to 1:593 as of September 2024, though still remains well below the ratios recommended by the American School Counselor Association (ACSA, 2003; 1:250), the National Association of Social Workers Association of America (NASW, 2018; 1:250), and the National Association of School Psychologists (NASP, 2020; 1:500) to move from exclusively crisis response to effective prevention and intervention systems resulting in desired positive school and student outcomes (Lapan et al., 2012; Stelmach et al., 2022).

The John H. Magill South Carolina School Behavioral Health Academy (SBHA) was created concurrently in 2022 with funding from SC DHHS to ensure newly connected school behavioral health professionals were prepared to work in schools and that districts and schools statewide were supported to collaborate with mental health professionals, families, and communities to provide comprehensive, efficient, and effective mental health support to young people, supporting functioning, well-being, and educational outcomes. SBHA advances the adoption and implementation of the Multi-tiered Systems of Support (MTSS) public health framework for prevention and intervention in schools. Numerous implementations of MTSS have demonstrated improved educational and behavioral health outcomes compared to school as usual in the United States, with PBIS being the most extensively and rigorously studied (Bradshaw et al., 2012, 2010). Implementations that include social, emotional, and mental health components have demonstrated superiority in access and outcomes over those that focus only on behavior (Cook et al., 2015; Splett et al., 2018; Weist et al., 2022).

To support and scale high-quality implementations of School Behavioral Health (SBH) MTSS, SBHA translates, disseminates, trains, and coaches individuals and organizations in best practices for collaboration, implementation, evaluation, and continuous improvement of both systems and supports. Program components include (1) self-paced, interactive online training, (2) customized coaching (implementation support) for district and school prevention and intervention teams, and (3) communities of practice to facilitate shared learning and evidence-based practice, including a regional annual conference. In this study, we describe the development of the SBHA coaching program and report on a qualitative evaluation of changes made by participating schools and districts, reported outcomes, potential mechanisms of change, and perceived coaching effectiveness determinants in South Carolina.

### 1.1. Development of a University-Based External Coaching Program

Implementing high-quality multi-tiered behavioral health supports is challenging, with leaders and teams requiring a strong support system of their own to achieve and sustain fit, fidelity, and outcomes (Freeman et al., 2017; Langley et al., 2010; Stephan et al., 2015). A recent systematic review of Consolidated Framework for Implementation Research (CFIR) constructs indicates that external change agents are one of eight key determinants identified as having the strongest impact on implementation across multiple project types (Schmitt et al., 2025). Positioned at a University inclusive of and connected to national and international leaders in the latest research and practice in school behavioral health (SBH), the SBHA team is ideally situated to serve as an external change agent in all aspects of its programming.

SBHA Coaching intentionally takes a community-centered systems coaching approach to develop the capacity of districts and schools to deliver a continuum of behavioral health promotion, prevention, and early and intensive intervention supports within an MTSS framework. Though somewhat varied in definition, systems coaching has been called for in both MTSS and SBH literature as essential to implementation (Fullan & Knight, 2011; March & Gaunt, 2013; Pierce, 2025). Chaparro et al. (2021) articulate systems coaching as the coaching of teams responsible for schoolwide implementation frameworks, such as MTSS. Pierce (2025) further describes systems coaching as a professional learning approach to improve the implementation of tiered frameworks. In addition to supporting teams in the implementation of MTSS, SBHA Coaching also addresses state and local cross-sector collaboration in educational professional practice settings, because school behavioral health services and preventive student supports are often delivered through community partnerships and public and private health care organizations. Key differentiation of SBHA Coaching from PBIS includes expanded teaming and collaboration, data collection and use, mental health expertise, and breadth and depth of evidence-based practice. Research on the nature, scope, and effectiveness of systems coaching in SBH is very limited, and this evaluation contributes to both program development and to our developing understanding of how, when, and why external systems coaching may “work” to support implementation.

### 1.2. SBHA Systems Coaching Components

SBHA Coaching is a collaborative process for systems change or improvement, guided by the Interconnected Systems Framework for connecting mental health and education systems, the National Center for School Mental Health Quality Guides, and the latest research in SBH across the globe (Eber et al., 2019; School Mental Health Quality Guides, 2020; Weist et al., 2023). Coaching is adaptive and focused on attainable community-defined goals related to SBH systems access and quality such as cross-sector collaboration; team startup, growth and sustainability; evidence-based practice training, coaching, and professional development; social, emotional, behavioral, and mental health screening and access systems; data-informed decision-making; and progress monitoring, evaluation, and continuous improvement systems.

#### 1.2.1. Coaching Structure and Process

District and school community leaders can connect with SBHA Coaching online through the program website (www.scsbha.org), via the program e-mail (sbha@mailbox.sc.edu), or through direct interaction with a coach or other faculty or staff member conducting outreach across the state. Districts and schools participate in the program at no cost, typically signing an agreement to participate in a systems inventory (described below), select and support school participation, and complete at least five coaching sessions across a full academic year. The SBHA Coaching participation agreement was created in response to district and school leaders expressing a desire for clearer structure and expectations around coaching, though it is not currently required to receive coaching support, and most schools and districts request and receive more intensive support. Five sessions were deemed attainable and appropriate to build new relationships and orient districts to our processes, though more is usually needed to support systems change. Coaching session content, dose, and duration were ultimately determined by goals set in the inventory process for most, with some adjusting their focus and goals over time.

#### 1.2.2. Tools

Coaching is designed and delivered based on needs and goals identified primarily through the completion of implementation inventories and a collaborative discussion of goals. Coaches offer high levels of direction and support based on experience and expertise and as needed by district and school teams. SBHA uses a slightly modified version of the District Systems Fidelity Inventory (DSFI; Kittelman et al., 2023) that includes an item related to well-designed mental health memorandums of agreement or understanding (MOAs/MOUs). The Interconnected Systems Framework Implementation Inventory (ISF-II) was selected as the most relevant and comprehensive existing inventory for schools (Splett et al., 2020). It was also modified somewhat to remove the assumption that schools were implementing PBIS already (most are not), to remove a single item perceived as inequitable for family engagement, and to add items documenting existing requests for assistance systems and the development and use of data decision-rules that were not previously captured. Changes were discussed with instrument developers and the SBHA coaching team. Both inventories cite original developers, and coaches are trained to request and crosswalk other MTSS inventories if they are in use for behavioral health. Additional coaching tools and guides from national implementation organizations are used by coaches and some are newly created or adapted by the SBHA team.

Coaches detail coaching topics, discussions, activities, and action items in notes taken for record-keeping and follow-up for each subsequent coaching session. Each district and school is unique in its systems needs and resources, and no single linear progression of coaching was prescribed. Coaching sessions ranged from 60 to 90 min, with most lasting 60 min. Some full-day coaching events were conducted in 60 min intervals for individual teams or cross-district audiences, including intervention teams, teachers, administrators, and school resource officers. Both in-person and virtual formats were offered. To date, two districts completed an inventory and then did not continue with coaching, citing a change in leadership that resulted in the loss of the leader that supported participation.

#### 1.2.3. Community of Practice

An annual “Coaching Institute” is hosted at the regional Southeastern School Behavioral Health Conference (SSBHC, see www.schoolbehavioralhealth.org) exclusively for district and school teams participating or expressing interest in participating in the SBHA Coaching program. This community of practice event fosters personal and professional relationships and facilitates shared learning for evidence-based practice. During the first annual event (held in 2024), participants were oriented to the implementation inventories and participated in small group discussion and large group sharing about their current approaches to SBH implementation. In the second year of the event (2025), attendees participated in semi-structured activities to develop their internal coaching roster and engaged in a panel discussion with veteran coaching schools and districts about their strengths and challenges. In the most recent event (2026), attendees engaged in cross-district and school discussion of three specific systems coaching topics (request for assistance systems, teaming, and resource mapping), guided by resources developed by the first author for the SBHA coaching program. As in the prior year, a panel discussion rounded out activities.

#### 1.2.4. Evaluation and Continuous Improvement

Current evaluation and continuous improvement efforts include training evaluation surveys and SBHA resource surveys, as well as the present qualitative formative evaluation study. Verbal and written feedback is used to make adjustments in real time and to plan future coaching. Program formalization and development are ongoing, and a coaching session survey has additionally been developed but not yet implemented.

### 1.3. SBHA Coaching Reach and Dosage

Since 2022, SBHA Coaching has worked with 14 school districts for a minimum of five contacts, initiating coaching on a rolling basis. Six districts and eight schools have completed at least one implementation inventory since their introduction in 2024. Five schools have completed at least 2 inventories documenting changes made in teaming, collaborative planning, family and youth engagement, intervention selection, implementation and progress monitoring, and data-informed practices. Between July 2024 and November 2025, 212 unique individuals from coaching districts completed SBHA self-paced courses in addition to team participation in coaching (Table 1). The low number completing the “Suicide Prevention for All” Course reflects that it was not released/available until October 2025. Because the program has been iteratively developed over time with participant feedback, the focus of the current study is not on reach or dosage of the developing program; rather, on participant experiences, which can inform the articulation of theories of change, program development, and further research.

### 1.4. The Current Study

Qualitative key informant interviews were conducted to answer three primary research questions:

(1) What changes were made and what outcomes were observed in districts and schools as a result of participation in SBHA Coaching?
(2) What feedback did participants have to improve SBHA Coaching?
(3) What were the barriers and facilitators of successful participation in SBHA Coaching?

## 2. Materials and Methods

This evaluation used a qualitative design to obtain rich, contextual understanding of participant experiences with and perspectives on the SBHA coaching program (Kazdin, 2022). This approach is well aligned with the research questions posed. The current study aimed to understand participants’ perspectives on how the program has been delivered and contributed to changes made and observed. The study was approved as exempt research by the Institutional Review Board at the University of South Carolina. We used the APA Journal Article Reporting Standards- Qualitative (JARS-Qual) to guide reporting (Levitt et al., 2018). Authors of this manuscript have formal education, training, and experience in education and/or developmental, clinical, and school psychology. Experience in school behavioral health research, theory, policy, and practice ranges from 2 to 30+ years. To the fullest extent possible, authors have worked to accurately represent implementation leader narratives, making use of extensive quotes to reduce bias and avoid speaking for participants. The first author is a research scientist with training in participatory methods and led the development of SBHA Coaching with co-authors and coaches. The second author and lead data collector is a doctoral candidate with firsthand experience providing MTSS coaching and consultation and implementing behavioral health interventions in schools. The third author served as second coder and is just beginning training in school psychology. Senior authors have extensive experience directing coaching, consultation, and the delivery of behavioral health support in schools. No authors served as primary coaches for SBHA schools and districts, and interpretations are the work of the authors.

### 2.1. Sample

District and school leaders receiving SBHA coaching support after the introduction of implementation inventories and a formal coaching process were invited to participate in evaluation interviews. Congruent with purposeful sampling, these two populations represent the individuals with the most direct experience with SBHA coaching and thus were ideally positioned to provide meaningful feedback on the program (Palinkas et al., 2015). A total of eight participants (*n* = 5 district leaders, *n* = 3 school leaders), 53% of those invited, accepted the invitation to participate, representing 80% of participating districts and 60% of participating schools. All districts and schools continue to participate in the coaching program. The final sample size is congruent with research suggesting thematic saturation of qualitatively analyzed individual interviews can be achieved in as few as six interviews (Guest et al., 2006). Participants represented a range of professional roles including district directors of student services and intervention (*n* = 2); district coordinators of well-being, clinical services, and MTSS (*n* = 3); a school principal (*n* = 1); an assistant principal (*n* = 1); and a school MTSS coordinator (*n* = 1). Participants were racially diverse (63% from communities of color) and predominantly female (75%). Table 2 summarizes characteristics of schools and districts represented and Table 3 summarizes coaching content, coaching participants, formats, and duration.

Participants were identified via established contacts with SBHA coaches. To protect participant confidentiality and promote open and honest feedback, a research assistant with no prior familiarity with any study participants conducted recruitment and data collection. The invitation letter emailed to all prospective participants included information about the study, the voluntary nature of participation, data protection, and participant incentives. All district leader participants received a $50 gift card to a vendor of their choice following the completion of their interview. School leader participants received a $30 gift card, with the reduced incentive reflecting the lower time commitment for school-level interviews.

### 2.2. Procedure

After reiterating information included in the invitation letter and obtaining verbal informed consent, participants participated in semi-structured, key informant interviews facilitated by a trained doctoral research assistant. Prior to data collection, two semi-structured interview guides were developed by the first and second author, one for district leaders and the other for school leaders. Questions examined participants’ roles and involvement in SBH implementation and their experiences working with their SBHA coach. Questions probed perceived changes in coaching targets, including teaming, organizational processes, and SBH practices within MTSS frameworks, as well as the factors (e.g., barriers and facilitators) influencing these changes across district and school contexts (Table 4). School leaders were asked about their work with district and SBHA coaches, as SBHA coaches work with district implementation leaders to intentionally develop capacity for internal district coaching to continue after external coaching support ends. Additional questions on the components of systems coaching (i.e., beyond evaluating the SBHA coaching program) were included in the district leader interviews for a separate study. Questions and findings from the separate study are not reported here. School leader interviews lasted 30–45 min, and district leader interviews ranged in duration from 45 to 90 min. Incentives were delivered electronically to all participants immediately following the completion of their interview.

All interviews were conducted, recorded, and transcribed using the Zoom teleconferencing platform (Zoom, 2025). Following each interview, the doctoral research assistant cleaned and de-identified each transcript. All data were stored on OneDrive, a secure server (Microsoft Corporation, 2025).

### 2.3. Analysis

A codebook approach to thematic analysis was employed to analyze content across interviews (Braun & Clarke, 2021a, 2021b). Codebook approaches to thematic analysis offer the flexibility to integrate inductive and deductive coding approaches focusing on constructing themes in response to specific inquiries (Braun & Clarke, 2021b; Byrne, 2022), as is the case in the present evaluation. Following codebook analysis procedures, a broad deductive framework aligning with the evaluation questions structured the coding process. Within this overarching framework, initial coding followed an inductive, constructivist approach, with two independent coders creating codes and themes based on participant responses. As the codebook developed, the coders used an increasingly deductive approach to organize new content into existing codes and themes as appropriate, while continued inductive coding was also permitted to generate new codes and themes. Thus, the codebook was iteratively developed and refined throughout the coding process.

The second author led the coding process, orienting and training the third author (and second coder). Coders spent time going through reflexive prompts to specifically consider how their backgrounds and prior experiences influence how they see the world and might impact coding. Discrepancies were normalized and a third independent coder process for resolving them was established in advance. In template analysis, it is expected that the codebook will constantly evolve over the course of coding, with the codebook continuing to evolve during the interpretation process. A memo/audit trail reflecting thought processes was created inside and outside the Dedoose coding system. Supplemental Table S1 shows cumulative new codes and saturation.

Coding followed the procedures for template analysis, which is a recognized approach to conducting codebook thematic analysis and generally aligned with the Braun and Clarke (2006) six-phase coding process (King, 2012). First, coders developed familiarity with the data by reading through the full data set at least once (Brooks et al., 2015; King, 2012). Then, they completed preliminary coding (i.e., first cycle or open coding), assigning descriptive codes—short words or phrases identifying a topic—to excerpts. Next, the coders created themes linking codes with similar meaning, through which process the initial codebook was constructed. The coders then applied the codebook to new data while simultaneously proposing new codes and themes (i.e., modifying the codebook) as appropriate. In the final step, the codebook was applied to the full set of data to ensure all data were ultimately analyzed using the same codebook. Coding followed a team-based approach to achieve greater depth of interpretation and enhance confidence in findings (Nowell et al., 2017). In accordance with standard reflexive codebook thematic analysis procedures, coding reliability metrics were not calculated (Braun & Clarke, 2021b). Rather, a consensus coding approach was followed, whereby the two independent coders met frequently throughout the coding process to review codes, consolidate overlapping concepts, and determine the final codebook used to identify and interpret themes. Discrepancies were resolved through consensus discussion and consultation with a third, independent contributor who was not involved in interview coding. All (100%) of coding disagreements were resolved through team discussion. Using methodology for determining thematic saturation described in Guest et al. (2020), 96% of themes were identified by the fifth interview.

Dedoose Version 10.0.34 was used to facilitate qualitative analysis (Dedoose, 2025).

## 3. Results

### 3.1. Outcomes

Codes nested under the Outcomes domain pertain to any changes participants reported having observed or made as a result of their participation in the SBHA coaching program. Perceived changes were analyzed and interpreted in five overarching themes: changes in knowledge, culture, administrative support, systemic structures and processes, and perceived impact of these changes. Thematic structure and exemplar quotes for perceived outcomes are presented in Supplemental Table S2.

#### 3.1.1. Knowledge and Awareness

Participants reported an increase in their own and their colleagues’ knowledge of SBH, including a better understanding of the framework, processes, and their role within the system. This included mental health literacy, greater awareness of the connection between behavioral health and academic outcomes, the importance of supporting students’ holistic development, and deeper understanding of MTSS beyond improving referral pathways. Five participants commented on how participation in SBHA coaching improved staff understanding of the tiered logic of MTSS, the need to provide universal support as well as targeted/intensive intervention, and how to match students to the appropriate intensity of support. A few participants also expressed that SBHA coaching enhanced their knowledge of specific SBH resources, including ways of continuing their learning and accessing additional resources. Knowledge was sometimes gained through SBHA coach interaction and modelling. As one participant described:

The teachers were so engaged in asking those questions and wanting to know how they could help. ‘Was it okay if I refer a student? What was their role? I know we have a school-based therapist, but I don’t know how to get to her, I don’t know how to get a child…’ Having [the SBHA coach] and her team come and really dispel some of those things and break down some of those barriers, that’s probably the biggest impact we’ve seen, with the awareness and being able to put the child in the support pathway that they need.

Participants also described taking the lead by verbally building knowledge and awareness in their own schools and systems without an SBHA coach present. An implementation leader described communicating tiered logic when called in to intervene:

And even when we have situations with the students, and I’m called in as MTSS, but the student is probably having behaviors that require Tier 3 support, I think I’m able to help walk them back through not pointing a finger at them, but let’s look at how our system, at how our processes could be improved at a Tier 1 level. So helping them navigate and work through, ‘all right, we gotta make a decision about this kid, but we need to consider this, this, and this.’ Versus just looking at what is happening with the kid, and let’s do something about that particular thing in that moment.

In the process of building awareness, this participant highlights “not pointing a finger at them”, using direction and divergent thinking as a driver of improved performance and student support.

#### 3.1.2. Change in Culture

Participants observed multiple changes in the culture of their districts and schools through their participation in SBHA coaching. Five participants perceived a shift in their school’s or district’s mindset toward behavioral health, noting that leaders and staff demonstrated a more comprehensive, holistic conceptualization of children’s and families’ needs and less resistance to integrating behavioral health into schools. As one district leader described:

Maybe we really need to be discussing not just academics. Maybe we do need to go back to what the original thought is—that if we’re going to do this, we’ve got to pull in a behavioral-mental health component as well. Because that’s really what happened last year. Curriculum instruction has taken over that MTSS process, and for them to then realize they’re not going to be able to really look at a child holistically if you don’t have people that are doing the work as part of those meetings. So now, they’re like, ‘oh, we need to be able to have a better vantage point.’

Multiple participants noted the development of a common language within their school or district that conveyed shared understanding of SBH. A participant framed their new approach to SBH as more “proactive” than it had been in the past:

And I’m finding, too, as the building leader, to make sure I keep that in my conversations, keep that in my daily flow when I’m doing the general morning message, or when we have a whole group faculty meeting, reminding them of what resources you have… I think one of my challenges, too, from this is to make sure we continue to bring awareness and not just when something happens, we become reactive. But we remain proactive with these resources, and if we have something free out there, use it, don’t let it sit there.

Participants also voiced a sense of greater interconnectedness amongst staff, including a sense of collaboration and cohesion, recognition that they are not alone, and that support is available for SBH.

#### 3.1.3. Administrative Support

Improvement in a few key types of administrative or organizational support was noted. Participants reported observing changes in how the district supports SBH implementation, such as increased site visits and meetings with school teams to provide consultation and improved bidirectional communication between schools and districts. A district leader describes listening to student support teams in schools and providing staffing support:

What I do is that, monthly, I go out to every single school in our district, including our alternative program as well as our intensive mental health program… We literally go to every school in our district. We sit down with every student support team meeting, myself and my director, and we listen to what has been going on that month. If they have things that they need to staff, like they’ve made it to us for services, this child’s in services, but still sending them more support. We talk about what we can do to provide them more support. That’s one of the things that I help implement district wide.

Two participants articulated specific changes in the district’s allocation of resources to support SBH, including establishing a new budget, protecting funds for SBH implementation, and increasing their SBH staff in schools to enhance SBH service delivery.

#### 3.1.4. Improved Quality of Systems

Improved quality of systemic structures and processes were frequently noted throughout the interviews. Participants reported perceived improvements in teaming, data-based decision-making, alignment and use of resources, professional development (including training and coaching), assessment and action planning, and community engagement through outreach. Many participants cited changes in teaming and collaboration. This included establishing and rebalancing the composition of teams, with participants specifically noting a shift toward more democratic power dynamics on teams.

You hear the thought process that all the decisions are made from the top down, by people who are not actually in the trenches in the field doing things. And one of the things that was very, very powerful and impactful I think was the fact that the SBHA coach called people to be part of teams that was a nurse, a bus driver, it could have been a teacher assistant. It wasn’t just administrators, and administrators had to actually take a back seat and listen to what their teams, from the school perspective of what’s going on… But I think that one of the things that was really powerful to hear… it was one meeting in particular where [staff] said, “we got a meaningful team” and it felt like ownership and being happy to be a part of a process that a lot of times they’re not included in. So that was definitely kudos to the SBHA process because it made everyone feel valuable. Their thoughts on different things were valuable as well. It didn’t feel like just directly being given out from the from the top down, but it felt like a community approach to improving.

Multiple participants reported improvement in the regularity of meetings and accountability for meeting attendance (particularly for school leaders). Less commonly, participants expressed improvement in communication and collaboration with families, vertical communication and upward advocacy from school to district teams and leaders, and efforts to redefine leadership roles to improve the consistency of implementation. External support functioned to open lines of communication as described by a district leader:

I think we have been able to open the line [of communication]. Our superintendent is fantastic. That is top-down. The communication level between our department and the top has definitely been better. We have been able to express our concerns and our questions and things that we want to see differently for our schools and get the support from the top down. That has been huge for us. And the coach has been a huge part of that, because things that maybe we really wanted to get across that maybe wasn’t being heard coming from the coaching side of it, and coming from the project itself to the higher-up was like, ‘oh, yeah, we probably should do these things.’ So getting that voice to be heard was really, really good.

As a result of their participation in SBHA coaching, school and district leaders reported an increase in the use of fidelity inventories to assess system-wide needs and identify critical gaps, establish goals and priorities, develop focused action plans, and evaluate progress. Relatedly, most participants described improvements in assessing/mapping, aligning, and using resources intentionally, enhancing the coherence of their SBH system. Data-based decision making—including thinking analytically, more frequent review and use of data, universal screening rollout, more streamlined and effective referral processes, and making procedures more explicit—likewise improved. Finally, districts enhanced their outreach efforts to make SBH personnel and resources more visible to the community and scaled up their professional development efforts, resulting in more staff being trained in SBH MTSS.

#### 3.1.5. Perceived Impacts

Participants perceived changes in more distal student/staff outcomes, which were often articulated in connection to the many improvements described above. Half of the participants specifically noted improvements in Tier 1 SBH programming (e.g., adoption of an evidence-based social–emotional learning curriculum or scaling up an evidence-based group intervention for universal implementation), and another couple reported increased attention to Tier 2 intervention. Some participants observed changes in student outcomes, with one reporting reduced behavioral issues and another commenting on an increase in referrals that they perceived to be related to greater awareness of student needs and available resources. Changes in teacher outcomes were also noted, including an increase in wellness initiatives for teachers and clinician support to teachers. As one participant described:

And we’ve got our therapists going in and modeling for the teachers, and modeling for the students the different behaviors and things, and for the teachers, the different interventions and the way to handle those particular students when situations arise… And I think they’re starting to finally realize they’re [the district is] not leaving me out here by myself. They are here to support, and if something’s not working, they’re willing to try something different with me, or give me other ideas and suggestions or point me in the right direction to get things that do work. And I think that’s been really big for the teachers, because, especially at the middle school level, where kids are not babies but not adults, and they’re all over the place, teachers did not feel any support a lot. And now they know you’re gonna have somebody come in and work with you. You’re gonna have somebody who you can reach out to to get that extra support that’s gotten these ideas and put these things in place and is willing to hear you and to push. And so I think that’s been a big change over the past 18 months is that they’re starting to feel that extra support within the school.

Participants perceived that leaders and staff felt more supported, motivated, engaged, committed, and hopeful about SBH as a result of changes made in relation to SBHA coaching.

### 3.2. Feedback

Codes organized under the Feedback domain are organized into positive or constructive feedback about the SBHA coaching program, including suggestions for improving the program. Many participants expressed positive feedback about the SBHA coaching program, including positive reception of and gratitude for coaching support. Participants reported enjoying the experience; finding the coaches engaging, responsive, and relatable; and appreciating the coaches’ nonjudgmental and collaborative approach, as well as the autonomy provided to districts to make decisions aligned with their priorities. Participants also expressed gratitude for the support and guidance coaches provide, noting that coaching has helped them connect to new opportunities and build capacity to continue the work more independently. Many of these topics are summarized by one participant, who shared:

I think that’s helpful, too, the fact that you all are very relatable. You have the background knowledge of what we’re going through. And then you’re just not saying, ‘oh you should do this,’ but you’re also willing to provide resources and follow up and help guide us. I think those are all great parts of coaching. I don’t really have any negatives to coaching… You provide us information, you equip us with some knowledge, and then we need to go and do. And some things are provided, some things are not, but it’s a good balance to equipping us to do and be better and not enabling us to take what you give us and say, ‘oh, it’s done.’

Finally, participants reported that coaching has helped them feel valued, expanded their perspective, and enhanced their motivation to advocate for needed change within their district. For instance, one participant stated:

Just having them on board has been a really great process. It’s causing me to think and work towards putting more information in place to make sure that things are there. It’s motivated me to advocate even more for mental health services and look at things a different way to make sure that the supports are there. So, it was really inspiring to see what work can be done… And I’m really grateful that our coach has been there and been supportive.

Constructive feedback, including suggestions for improvement, was mentioned slightly less frequently. The most common suggestions were to expand coaching to more schools and increase training. Regarding expanding school-based coaching, participants suggested coaches increase the number of schools they work with and follow up more frequently with school teams. Two participants specifically requested an increase in site visits or learning walks to observe practices in real time, and one participant suggested that schools may benefit from a more directive approach with explicit guidance on the sequence of actions to take. The desire for increased coach time on site also emerged with respect to training, as multiple participants requested an increase in in-person training.

Additional points of constructive feedback pertained to a desire for support for district administration and teams, opportunities to better translate information by reducing the use of terminology and re-framing information to ensure it is digestible for all coaching participants, and requests for the SBHA to engage in state-level advocacy, as poor state guidance and support was noted as a barrier to higher-order change. Finally, participants suggested enhancing reciprocity by offering continuing education credits for participating in coaching and/or in-person professional development delivered by coaches and providing participant incentives to maintain engagement and display appreciation.

### 3.3. Barriers and Facilitators

Barriers and Facilitators include themes and codes that pertain to factors that impede success and factors that enable or enhance success, respectively. Barriers and facilitators were coded with respect to the coaching relationship (i.e., factors that interfered with or facilitated coaching) and perceived coaching impacts (i.e., factors that impeded or enhanced the school’s/district’s ability to benefit from coaching). Given substantial overlap in concepts, barrier and facilitator themes and codes were collapsed across categories to summarize the factors participants perceived to influence the success of the SBHA coaching program. Two broad themes were constructed: coaching factors, which include characteristics of the coach and the coaching relationship, and organizational factors, which include district/school infrastructure, norms, mindsets, and knowledge that influence the success of SBHA coaching.

#### 3.3.1. Coaching Factors

Participants highlighted the role coach characteristics and skills play in enabling them to benefit from coaching. These characteristics and skills include communication patterns, with participants noting that communicating clearly to set explicit expectations about what coaching and SBH implementation entail and maintaining consistent contact with frequent follow-up facilitated coaching success. Coach knowledge and experience were identified as similarly important to success. Specifically, participants expressed that the coach being well-informed about events and trends in education and the greater society and the coach having prior experience working in schools were integral to success. As one participant put it:

They’ve got to know the people that they’re working with. I think that’s huge. I think they need to be competent in the area that they’re coaching in. If I’ve got somebody coming in to coach, I don’t want a basketball coach coaching football, so I need to know that whoever’s coming in truly understands behavioral health and how that works within a school setting. And what the needs of that building are, not just understanding the audience, but the needs itself.

The ways coaches approach and build the coaching relationship were also underscored. Many participants commented on the collaborative nature of the coaching relationship, repeatedly referring to it as a “partnership” characterized by trust and psychological safety that laid the foundation for coaches and recipients to have forthright conversations about districts’ needs, priorities, and realistic pathways to change. Coaches’ responsivity—including being accessible, available, and often present in person—and their orientation toward capitalizing on strengths and aligning their efforts with districts’ existing initiatives set the conditions for efficient and effective change efforts. A district leader described:

I think it was empowering us to see what we already had in place, because usually when you have a program come in, they come out with ‘everything’s going wrong.’ That was not the process that was followed by our coach. Our coach helped us to realize what we already had in place to perfect those processes and then implement it in an effective way, so we didn’t just have a bunch of things and have all of these mediocre processes, but just being able to utilize what we have, to be able to streamline those systems.

Participants further identified a number of coaching approaches and methods that were particularly instrumental in facilitating change with SBH implementation. Participants found value in training coaches provided to leaders, teams, and school staff on a variety of SBH topics, especially when delivered in person. Participants also spoke about coaches’ use of reflective, open-ended questions that facilitated deeper engagement with the implementation/change process, coaches’ ability to connect districts and schools to new resources, and the support coaches provided with data analysis and interpretation as facilitators of SBH implementation. Of all of the coaching methods identified, needs and resource assessment—carried out through the collaborative completion of system-wide SBH fidelity inventories and resource mapping activities—was the most frequently articulated as consequential for promoting change. As one participant put it:

It actually was the metrics, like the actual inventory. Giving that data and that information directly to the curriculum instruction and saying, ‘this is what [District Leader] and I see, but it’s not all-encompassing,’ but literally gave them and sent it to them. And that’s when they began to, over the summer, figuring out we are not using the right approach, we’re not using the evidence-based strategy. I didn’t realize that we actually have people that can help guide, and also there are templates, and we’re making things up on our own. So that inventory literally, actually, by completing that last year, gave a thrust to say we did not prioritize behavioral mental health.

In addition to systems assessment and data-based decisions, another participant highlighted support for both short- and long-term intervention goals:

And then realistic interventions, interventions, realistic skills, realistic tips, things that we can implement quickly. And then other things that take a little longer, right? We need some quick fix, and we need some things that we can slowly roll out throughout the year. I think they did a good job with that with us, too, as things that were instant that we could work on, and then things that we had to bring back, or they had to bring back to us from those meetings and sessions to things that we could work on and roll out throughout the year.

Both coach characteristics and instrumental and practical tools were interpreted as significant and influential to participants. Thematic structure and exemplar quotes for coaching factors are presented in Table S3.

#### 3.3.2. Organizational Factors

Organizational factors related to district infrastructure (i.e., existing resources or lack thereof) encompassed the greatest number of codes. Participants identified time, funding, and staffing as key factors that can either promote SBH implementation (if present in sufficient quantities) or undermine it (if absent). Participants articulated how competition between district priorities and initiatives over these finite resources, as well as district policies and procedures, constrain or slow SBH implementation. For instance, one participant shared:

I think the barriers for us are sometimes district protocols that we have to abide by. For example, making sure the legal attorneys look over a study that we want to participate in. Or the tech team, make sure that what we want to do with rostering takes place. Most of the time, the barriers are some of those limitations that we just don’t have control over that. They’re just district things that have to be done due to privacy or different legislative mandates that are out… And time, time is always a factor, working with schools and trying to figure out scheduling, and a lot of that is mandates. For example, in our state, third grade, Read to Succeed, if they’re not reading on grade level by the end of the third grade year, they’re retained. That’s a big priority. So, telling that to principals when we’re talking about, “well, this is what we have to do with mental health,” but they’re focused on, “we don’t want to have all of these third graders retained.” It’s just making sure that we have that balance. I think that’s barrier that just hits us, that sometimes things, regulations, get in the way of the other pieces.

Other participants identified community resources such as local wellness initiatives and having behavioral health providers in the area as helpful for improving SBH implementation, or interfering with implementation when communities are under-resourced. Finally, participants addressed the importance of having strong leadership support that goes beyond buy-in, as well as access to high-quality data, to both participate meaningfully in coaching and to achieve desired change in SBH implementation.

In addition to infrastructure, participants articulated various norms, mindsets, and pieces of knowledge that influence participation in and the impact of SBHA coaching. With respect to norms, a culture that promotes collaboration, emphasizes accountability, and is characterized by trust within teams and between school/district leaders and staff facilitates change. As one participant said, “I think making sure that the leader, the school, the staff, they’re in a place where they can trust each other to be vulnerable. They have some honest conversations, because the relationships in my building are varying levels.”

On the other hand, participants noted that rigid top-down leadership hierarchies, competitive dynamics between staff, and poor collaboration with families and community undermine SBH implementation. In terms of mindsets, participants expressed that recognizing a need for change, openness to change (or willingness to do things differently), investment in change (i.e., “buy-in” to SBH), and perceived capability to engage in or achieve change enabled their schools/districts to benefit from coaching. Explaining a sense of capability, one participant noted:

“So I’m not necessarily saying that you’re willing, that wouldn’t be the biggest part of it, but certainly a part of it, but more so when I say ready, I mean… that there’s an opportunity for alignment with what I’m providing. So there’s an opportunity, there’s a need, but then there’s something in your personality, or something in your demeanor, or processes, or system that says, ‘I’m capable…’”

Investment in change, often phrased around “buy-in” to SBH and/or coaching, was the single most frequently articulated factor that participants perceived to influence outcomes. In particular, many participants spoke to the importance of district and school leaders being invested in SBH. Lastly, schools’ and districts’ baseline knowledge of SBH was noted to influence outcomes, with an existing understanding of MTSS and the interconnectedness of academic and behavioral health outcomes noted to be beneficial. Thematic structure and exemplar quotes for organizational factors are presented in Table S4.

## 4. Discussion

Feedback on the SBHA Coaching Program was overwhelmingly positive and actionable. Changes made in districts and schools encompassed leadership and administrative support, systems practices, and SBH and MTSS knowledge, as expected and directly targeted by SBHA coaching activities. Surprisingly, reported outcomes also extended to perceived changes in culture and impacts on student and teacher well-being, which are generally considered longer-term outcomes of systems change efforts.

Barriers and facilitators were often equally expressed within themes, although some were mentioned more as one or the other (a barrier or a facilitator). A notable example is that more codes were generated tied to norms in opposition to recommended practice (i.e., hierarchy over collaboration, ego over collective goals). One interpretation is that opposite or contrasting norms are more salient because they significantly limit the change process, representing a strong potential effectiveness determinant. Innovative coaching approaches and research that assesses and implements evidence-based social norm or values activating interventions in systems coaching contexts would be beneficial (e.g., Cislaghi & Berkowitz, 2021). In contrast, mindset codes were largely reported as facilitators, including positive motivation for change and capability attributed to coaching program participation, which are key mechanisms of change identified in both behavior change and organizational change models and literature (Michie et al., 2011; Domlyn et al., 2021). Many systems theorists identify mental models as foundational for change (Schaffernicht et al., 2025). Changes in mental models may alter relational dynamics, which may then result in changes in organizational structures and policy. Change approaches that begin with structure and policy may be less sustainable if those changes are easily changed and discarded, for example with a change in leadership or with widespread opposition. Coaches who are able to shift mindsets may be particularly transformational.

Responses support evidence from an extensive body of literature on the importance of leadership in change and implementation. Participants referenced leadership broadly, sometimes formal leadership roles like superintendents, principals, and other administrators, and other times in reference to SBH or MTSS implementation leadership. Administrative support was described as instrumental (resources and policy), procedurally supportive, inspiring, or needed across different contexts. Corbin et al. (2024) provide evidence suggesting strategic implementation leadership is tied to lower burnout, but transformation leadership is not, underscoring the need for both to serve different purposes. Implementation leaders for specific implementation projects are also identified as a key determinant of implementation outcomes (Schmitt et al., 2025).

Coaching skill facilitators included communication, content knowledge, experience, and relationship skills. Key approaches and methods identified as helpful (e.g., reflective questioning, needs assessment, goal setting) are similar to those found in teaching, counseling, and other helping professions, suggesting professional expectations of systems coaches should align with formal training in other helping professions. Systems assessment and resource mapping were overwhelmingly identified as catalysts for change.

### 4.1. Theory Development and Future Directions

Findings provide support for the perceived influence of SBHA Coaching in promoting the implementation of SBH within an MTSS framework and the potential for producing expected downstream staff and student outcomes. Participants articulated potential mechanisms of change that align with the capability, opportunity, and motivation model of behavior change (COM-B; Michie et al., 2011), which may represent a theoretical extension of the model to complex systems change. Implementation leaders highlighted barriers and facilitators along all three pillars of Fixsen and Blase’s (2008) Implementation Drivers Framework: competency, leadership, and organization. In the Implementation Drivers Framework, the three dimensions are ideally integrated, but they can be compensatory if one area is weak, underscoring a need for adaptability in systems change interventions and for systems assessment at the start of any change effort. Indeed, participants pointed to system inventory metrics as a starting point for recognizing the need for change and for selecting targets of change efforts.

Competency and organizational drivers in the Implementation Drivers Framework lead directly to implementation fidelity of evidence-based practice, the hypothesized mechanism of change for positive student outcomes. We view SBHA Coaching as a systems intervention concerned with supporting fit and fidelity of empirically supported practices across the tiered continuum of behavioral health promotion, prevention, and intervention, as well as fit and fidelity of the systems that support their delivery. Personnel, community collaboration, and evidence-based practice are markedly enhanced in SBH MTSS in comparison to Positive Behavior Interventions and Supports (PBIS), but implementation support and systems practices are highly similar (i.e., an MTSS framework). Systems intervention is illustrated as an organizational driver in the Implementation Drivers Framework, but our findings suggest that the SBHA Coaching model may impact leadership and competency as they relate to SBH systems as well, extending application of the framework beyond singular evidence-based programs and practices. Participants referenced psychological and systems change theory constructs frequently, such as relationship dynamics between coaches and coachees and among district and school team members, and mindsets toward SBH and change, which strongly merit study in SBH systems change efforts involving both individual and group behavior.

Finally, state, district, and school policy alignment supporting behavioral health MTSS could significantly impact outcomes for communities and young people. As observed by Albers et al. (2022), implementation support is an infrastructure investment. A strong evidence base is needed to guide implementation support programs, including rigorous effectiveness studies. Future program development and research goals include model formalization, standardization, and testing that retains adaptable, strengths-based, and community-centered features. Process evaluation that carefully tracks systems change, not only static start and end points, has the power to more comprehensively illustrate complex systems change (McGill et al., 2020). Dose–response studies could further illuminate how effective SBH systems coaching is under common conditions described by participants, for example when academic initiatives take precedence or leadership turnover is high.

### 4.2. Limitations

While study strengths include credible capture of breadth and depth of experiences in the SBHA program, there are several limitations to this study. First, coaching program interviewees voluntarily participated in the SBHA coaching program, so interviews did not capture the views of those who were unable to fully participate, likely for reasons related to coaching effectiveness determinants. Our formative internal evaluation focused on the perceptions of those engaged in coaching services to some extent. Although this illuminated barriers and facilitators in the coaching context, it did not capture initiation or engagement failure. Second, reports were provided by implementation leaders who may have a positivity bias compared to implementers in the same school or district. Lead implementers may also have social desirability biases linked to positive collegial relationships with coaches and others at the University. Community engagement is central to the coaching model, SBH research, and the mission of the University (e.g., Figas et al., 2025). External evaluation is needed to contribute convergent and divergent evidence. Future work should capture the perspectives of multiple informants, including implementers (e.g., school psychologists, social workers, teachers), staff, students, and those unable to fully participate in coaching, who may have critical insights for coaching systems development, including regulatory and policy needs around access. Quantitative measurement of staff well-being and student social, emotional, behavioral, and academic outcomes and rigorous causal design is needed to fully examine links between systems coaching, implementation, and individual and public health outcomes.

## 5. Conclusions

Implementation support models for public health frameworks for intervention (e.g., MTSS) are increasingly funded by state agencies and institutions of higher education but remain understudied. By articulating program components and presenting themes constructed from qualitative key informant interviews on SBHA Coaching, our findings support systems change theory development for SBH and testable hypotheses potentially linking specific external coaching relationship characteristics, processes, skills, and activities to changes in SBH awareness, culture, systems, implementation, and staff and student outcomes. Behavior change theories and the Implementation Drivers Framework may be useful for articulating theories of change for SBH systems coaching models, though the breadth and depth of qualitative findings support the need to further develop systems change theories and frameworks (e.g., key mental models, relationship dynamics, and systemic structures), some of which are articulated, for example, in guides for understanding and implementing the Interconnected Systems Framework (e.g., Barrett et al., 2019). By explicitly incorporating systems change theory into design and delivery, SBH implementation support approaches have the potential to result in sustainable change. Rigorous quantitative and mixed methods work is needed to formally test, complement, and extend the findings of this study, moving the field toward evidence-based implementation support for SBH systems.

## Figures and Tables

**Table 1:** Coaching Participant Completion of SBHA Self-Paced Courses.

Course Title	Personnel in Coaching Districts
All Hands on Deck	94
Core MTSS Practices	101
Building the Foundation with Tier 1	71
Bridging Supports with Tier 2	63
Growing Your Tier 3 Supports	136
Elevating Family Partnerships	40
The Opioid and Fentanyl Epidemic	18
Weaving the Tapestry: Suicide Prevention for All	3

**Table 2:** District and school characteristics.

	Locale (NCES Designation)	School Type
District 1	City, Small	
School 1 (District 1)	City, Small	Elementary (PK-5)
District 2	Town, Fringe	
District 3	City, Small	
School 2 (District 3)	Suburban, Small	Middle School (6–8)
District 4	Suburban. Large	
School 3 (District 4)	Suburban, Midsize	Combined
District 5	City, Small	

*Note*. NCES = National Center for Education Statistics, PK = Pre-kindergarten; NCES designations retrieved using the NCES Locale Lookup Tool: https://nces.ed.gov/programs/maped/LocaleLookup/ (accessed on 22 July 2026).

**Table 3:** Summary of customized coaching topics, participants, formats, and total duration.

	Topics	Participants	Format	Hours
District 1 + School 1	Screening and data-based decision-making Tier 1 and Tier 2 practices	District implementation lead and school team	In-person	2 h
	Tier 2 planning MTSS meeting scheduling Teaming Understanding internalizing concerns Collaboration with support partners	School team	In-person	15 h
District 2	Resource mapping MTSS guidance document creation and review PD and training systems Professional learning communities	District team	In-person	4 h
District 3 + School 2	Universal screening Needs analysis Evaluation and PD planning	District team	Virtual In-person	4 h 1.5 h
	Request for assistance process Tiered intervention system	Teachers	In-person	6 h
	Progress monitoring Intervention planning	Cross-district intervention staff and teachers	In-person	6 h
District 4 + School 3	District coaching planning Teaming Training with the LMS Completing and using inventories Universal screener selection and readiness to use Progress monitoring Teacher buy-in Tiered intervention system	District implementation lead and school team	Virtual	5 h
District 5	Leadership and teaming Community member engagement Use of Title 1 funds Public outreach Funding and alignment for Tier 1 and Tier 2 Medicaid billable services for Tier 3 Substance use prevention and intervention programming Policy changes to support staff training and delivery of services	District Team	In-person	3 h
	Request for assistance system Tiered intervention system	Cross-district intervention staff and teachers	In-person	6 h

*Note*. PD = Professional Development, LMS = Learning Management System.

**Table 4:** Interview Guides for District and School Leaders.

District Leader Questions	School Leader Questions
How would you describe your current involvement with SBH Systems Coaching [your SBHA coach]? How would you describe your current role in your district related to SBH? Probes: What is your title? What do you do to support SBH implementation? How long have you worked with a SBH systems coach [your SBHA coach]? How would you describe the role of your SBH systems coach [your SBHA coach]? What changes have you seen or made, if any, as a result of your interactions with your Academy [SBHA] Coach? Prompt: Any changes in district teaming or organizational processes? Prompt: Any changes in your relationships/activities with schools? Can you describe any formal or informal guidance you have provided to schools to support the implementation of school behavioral health within MTSS? What changes have schools in your district seen or made, if any, as a result of interactions with your Academy [SBHA] coach? Prompt: Any changes in school teaming or organizational processes? Prompt: Any changes in implementation of the LMS, inventories, or school behavioral health within the MTSS framework? From your perspective, what factors contribute to a successful SBH systems coaching experience? Provide examples if needed: characteristics of individuals involved, school/district context, external systems, etc. What challenges or barriers get in the way of your systems [SBHA] coach being able to provide the support your district needs to implement SBH? Provide examples if needed: characteristics of individuals involved, school/district context, external systems, etc. What needs to occur or be in place for SBH systems coaching to be effective? What could your systems [SBHA] coach do to better support your district with SBH implementation? Is there anything else you would like to share about your experience or perspective on SBH systems coaching?	What is your role in your school? When did you start working with your District and SBHA coach? What changes has your school seen or made, if any, as a result of interactions with your District and Academy Coach? Prompts: Any changes in school teaming or organizational processes? Any changes to implementation of school behavioral health systems and practices within the MTSS framework? What specific factors do you think were involved in the above changes? What needs to be in place for coaching to be effective/helpful to you?

## Data Availability

The original contributions presented in this study are included in the article/supplementary material. Further inquiries can be directed to the corresponding author.

## Supplementary Materials

The following supporting information can be downloaded at https://www.mdpi.com/article/10.3390/bs16081391/s1. Table S1: Thematic Saturation; Table S2: Thematic Structure—Perceived Outcomes; Table S3: Thematic Structure of Barriers and Facilitators—Coaching Factors; Table S4: Thematic Structure of Barriers and Facilitators—Organizational Factors.
