# Supplementary material for: Development and Evaluation of a University-Based External Coaching Program for School Behavioral Health
Source: Behav Sci (Basel). 2026 Aug 13;16(8):1391. doi: 10.3390/bs16081391 (PMC13509704; doi:10.3390/bs16081391)
Supplement: Supplementary file 1 [file behavsci-16-01391-s001.zip › behavsci-4323269-supplementary.pdf]

Supplemental Table S1

Thematic Saturation

Interview	Codes Applied (n)	New Codes Created (n)	Total Codes in Codebook (n)	Percent New Information	Percent Saturation
Interview 1	65	0	65		
Interview 2	50	13	78		
Interview 3	40	7	85		
Interview 4	40	5	90		
Interview 5	43	4	94	4.44%	95.56%
Interview 6	21	1	95	1.06%	98.94%
Interview 7	40	3	98	3.16%	96.84%
Interview 8	36	3	101	3.06%	96.94%

Note. Saturation was calculated using the method detailed by Guest and colleagues (2020). To calculate saturation, the base was set at four interviews (90 codes). Run length was set at one interview given the small sample.

Supplemental Table S2

Thematic Structure- Perceived Outcomes

Theme	Subtheme	Exemplar Quotes
Knowledge and Awareness	SBH MTSS	*"The teachers were so engaged in asking those questions and wanting to know how they could help. 'Was it okay if I refer a student? What was their role? I know we have a school-based therapist, but I don't know how to get to her, I don't know how to get a child...' Having [the SBHA coach] and her team come and really dispel some of those things and break down some of those barriers, that's probably the biggest impact we've seen, with the awareness and being able to put the child in the support pathway that they need."*

	Tiered Intervention	*"And even when we have situations with the students, and I'm called in as MTSS, but the student is probably having behaviors that require Tier 3 support, I think I'm able to help walk them back through not pointing a finger at them, but let's look at how our system, at how our processes could be improved at a Tier 1 level. So helping them navigate and work through, 'all right, we gotta make a decision about this kid, but we need to consider this, this, and this.' Versus just looking at what is happening with the kid, and let's do something about that particular thing in that moment."*
	SBH Resources	*"But turns out the district had already done those things, we just weren't aware. So that helped open our eyes to other resources that were available."*
Change in Culture	Mindset toward BH	*"Maybe we really need to be discussing not just academics. Maybe we do need to go back to what the original thought is—that if we're going to do this, we've got to pull in a behavioral-mental health component as well. Because that's really what happened last year. Curriculum instruction has taken over that MTSS process, and for them to then realize they're not going to be able to really look at a child holistically if you don't have people that are doing the work as part of those meetings. So now, they're like, 'oh, we need to be able to have a better vantage point.'"*
	Common Language	*"And use some common language with our principals, with our teachers. Even our school counselors, I will say, I don't think we're as trained for mental health needs as I probably would have thought that they would have been. It's just, let's let someone else do it, but it has helped us to think, we need to make sure everyone is speaking the same language."*

	Interconnectedness	*"And we tell the teachers like we're all learning and working together. You can't get this wrong. There's no right or wrong way to do this, but let's just walk hand in hand. Then we're going to get this done."*
	Proactivity	*"And I'm finding, too, as the building leader, to make sure I keep that in my conversations, keep that in my daily flow when I'm doing the general morning message, or when we have a whole group faculty meeting, reminding them of what resources you have...I think one of my challenges, too, from this is to make sure we continue to bring awareness and not just when something happens, we become reactive. But we remain proactive with these resources, and if we have something free out there, use it, don't let it sit there."*
Administrative Support	Budget	*"Having the coach come on board, I feel like helped impact my team be able to have a budget to be able to go to more trainings, to be able to implement, to have the resources to buy more therapeutic resources to utilize in their offices. My team haven't had a real budget ever. We got our first account number maybe six or seven years ago. There was an account, but it only covered ink and occasionally a couple of board games. Now, our coach helped put that in place, so every therapist got $500 worth of resources that they can utilize in their offices and be able to help the students and they're so excited."*
	Staffing	*"I think one of the things that maybe has changed is now we have the school-based therapists in every school."*
Quality of Systems	Team Formation and Composition	*"You hear the thought process that all the decisions are made from the top down, by people who are not actually in the trenches in the field doing things. And one of the things that was very, very powerful and impactful I think was the fact that*

	the SBHA coach called people to be part of teams that was a nurse, a bus driver, it could have been a teacher assistant. It wasn't just administrators, and administrators had to actually take a back seat and listen to what their teams, from the school perspective of what's going on...But I think that one of the things that was really powerful to hear... it was one meeting in particular where [staff] said, "we got a meaningful team" and it felt like ownership and being happy to be a part of a process that a lot of times they're not included in. So that was definitely kudos to the SBHA process because it made everyone feel valuable. Their thoughts on different things were valuable as well. It didn't feel like just directly being given out from the from the top down, but it felt like a community approach to improving."
Team Functioning and Accountability	*"We have student support team meetings and they happen every week in every school. And the teams have the responsibility of tracking their meetings and talking about what do the kids need and then what services that they need to provide. And you have administrators around the table. You have the school counselors and the school-based therapists, as well as our contracted services are also around that table every week talking about, 'this kid is coming across my desk. This is what's going on.' And they are able to discuss who's going to step in to help this child."*
Role Redefinition	*"What we found was that there was some inconsistencies with how behavior was being handled with splitting the grade levels. And we also found that because the other assistant principal had a larger focus on academics, that they weren't able to have their thumb on behavior like I'm able to now. So we made that transition to me being the sole person that looks at the behavior, school life. It's worked out nicely."*

Vertical Communication	*"I think we have been able to open the line [of communication]. Our superintendent is fantastic. That is top-down. The communication level between our department and the top has definitely been better. We have been able to express our concerns and our questions and things that we want to see differently for our schools and get the support from the top down. That has been huge for us. And the coach has been a huge part of that, because things that maybe we really wanted to get across that maybe wasn't being heard coming from the coaching side of it, and coming from the project itself to the higher-up was like, 'oh, yeah, we probably should do these things.' So getting that voice to be heard was really, really good."*
Family Partnerships	*"Working with our coach really helped us realize that, one ,we need to do better with communicating with parents about where their student is. Like we would meet with parents, but we wouldn't say, 'hey, your kid is here, too. This is the interventions they're going to go through and we're going to follow up.' We really didn't follow up unless there was an issue. And so that's something that we want to work on as well. We're slow and steady right now, but we're getting there."*
Procedures	*"They have also helped us recreate the emergency card. It says what to do if the student has an issue, what track to follow, when to contact the school counselor, the school-based therapist, the administrator. They've redone that part with us as well, so that there is a tracking for them to find with that."*
Universal Screening	*"Our district was one that was on the fence a little bit with the universal screener because you're going to have alerts everywhere. Then, finally, in year 2, from my supervisor and the powers that be, finally did say yes to advocating for those so you can be able to screen and then now we're on board with*

	doing universal screening for this school year. She [SBHA coach] has been instrumental with showing the information that you gather as a result of those screenings."
Referral Processes	*"As a result of that, we saw better referrals for our children. The teachers are now able to identify some different signs, or they pick up on things, and they're quick to communicate, whether it be with their grade-level administrator, a school counselor, or directly with the school-based therapist, to make sure that the supports are in place."*
Use of data	*"I feel like we're more structured and we are becoming more data-driven regarding behaviors and looking at behaviors, really trying to categorize behaviors and weed out who's truly a Tier 2 student versus a Tier 3 student and developing that process."*
Assessment and Action Planning	*"Another layer that was eye-opening, when we did the inventories. Certain things we thought we were doing, or we had in place, we realized we weren't doing with fidelity. Or, we said we had it, but do we really have it? We're still working on strengthening our Tier 1 supports. We have great supports for Tier 3...That's one of the things we're still working on. We were able to identify that gap as a result of having some real conversations."*
Alignment and Use of Resources	*"Our coach helped us to realize what we already had in place to perfect those processes and then implement it in an effective way, so we didn't just have a bunch of things and have all of these mediocre processes, but just being able to utilize what we have, to be able to streamline those systems."*
Professional Development	*"We've had more teachers and administrators over the last couple of years experience trauma-informed care training.*

		They've had more teachers and more administrators involved in that, and that has helped become a part of all the other things that add to the conversations and the processes."
	Outreach	*"We felt like that was a strong place, and now, I'm proud to say, on our district website, we have a Department [name masked] page, we have all of our social workers listed with their pictures. All of our [rehabilitative services] people list them with their pictures, who we contract out with, and the resources that we have in our district, so it's a one-stop shop where you can go. Because one of the things I think that maybe came up in that survey [inventory] wanted to make sure we increase visibility for all of our partners."*
Perceived Impact	Improved EBP Delivery	*"Perfecting our tier one processes. That is one of the major changes. A child is struggling in school, in the classroom and immediately calling for administration is something that does happen often. And because of the different things that have been implemented because of the SBHA whole process, we've been giving them the tools back in their hands to be able to utilize what you have. That was one of the major things that has happened as a result of having the processes in place."*<br><br>*"They've actually really honed in on their Tier 2 services. They're realizing that they really didn't understand exactly how to implement Tier 2, and they're working through that."*
	Student Wellness and Support	*"Now we're showing there's actually measures that we are able to show the decrease within certain behaviors, and also because of some of the emotional processing and things that we've been teaching, we are seeing a decline in certain classrooms."*

Teacher Wellness and Support	*"And we've got our therapists going in and modeling for the teachers, and modeling for the students the different behaviors and things, and for the teachers, the different interventions and the way to handle those particular students when situations arise...And I think they're starting to finally realize they're [the district is] not leaving me out here by myself. They are here to support, and if something's not working, they're willing to try something different with me, or give me other ideas and suggestions or point me in the right direction to get things that do work. And I think that's been really big for the teachers, because, especially at the middle school level, where kids are not babies but not adults, and they're all over the place, teachers did not feel any support a lot. And now they know you're gonna have somebody come in and work with you. You're gonna have somebody who you can reach out to to get that extra support that's gotten these ideas and put these things in place and is willing to hear you and to push. And so I think that's been a big change over the past 18 months is that they're starting to feel that extra support within the school."*
Expansion	*"K through 8, we do a pretty good job of MTSS. The elementary schools, K-5, pretty much use PBIS with their MTSS system. But 9 through 12, which is where we've started pushing in last year, and there was some pushback, but now this year, full force."*
Motivation and Commitment	*"It's motivated me to advocate even more for mental health services and look at things a different way to make sure that the supports are there."*
Hope/Vision	*"It's helped me and my supervisor feel there's a path forward."*

Supplemental Table S3

Thematic Structure of Barriers and Facilitators- Coaching Factors

Theme	Subtheme	Exemplar Quotes
Characteristics and Skills	Clarity	*"And being open, transparent, this is hard work. You're looking at a system that has been doing things a certain way, and now we're asking you to branch out and bridge and think about things in a whole different mindset. Being honest about that. [SBHA Coach] has done that."*
	Consistency	*"We've met regularly over 18 months, constant meeting."*
	Knowledge and Experience	*"They've got to know the people that they're working with. I think that's huge. I think they need to be competent in the area that they're coaching in. If I've got somebody coming in to coach, I don't want a basketball coach coaching football, so I need to know that whoever's coming in truly understands behavioral health and how that works within a school setting. And what the needs of that building are, not just understanding the audience, but the needs itself."*
	Collaborative Relationship	*"I think the most successful one is when you have that relationship with them, and they know that you're here to work, they're here to work with you, and not work against you. Like, we're working really as a team. That creates the most effective experience."*
	Strengths-Based	*"I think it was empowering us to see what we already had in place, because usually when you have a program come in, they come out with 'everything's going wrong.' That was not the process that was followed by our coach. Our coach helped us to realize what we already had in place to perfect those processes and then implement*

		it in an effective way, so we didn't just have a bunch of things and have all of these mediocre processes, but just being able to utilize what we have, to be able to streamline those systems."
	Trust-Psychological Safety	*"I think working with our coach in particular. I felt like she heard us. It wasn't a judgmental thing like, "y'all messed up." It was never anything like that or "y'all should be doing..." It was never anything like that. So I think feeling safe with the coach and feeling like we were in a judgment-free zone and that she really wanted to hear us and then help us. I was a huge fan after."*
	Responsiveness	*"She came in and met with a smaller team to introduce the process and from then on she answers every single e-mail...In order to have buy in, we have to start with our principals and she came in and had that full meeting with them. She answered all of their questions and then she also let them know that we're working hand in hand so that as they have questions and things as it works from the district level, they had me and they also had her and she was responsive to all the questions at different junctures in the process."*
	Alignment with Existing Efforts	*"First of all, let me say that the coaches right up front establish a partnership with me to help me serve my schools with their services in line with what we had in place."*
Coaching Methods	Training	*"They went into our PLCs in our middle schools, and actually met with all of the teachers in the middle schools. And then they also met with all the administrators in the middle schools. And then they've met with the counseling teams in the middle schools. So, it's not just a one-on-one or a one-size-fits-all for everyone. It was definitely going in and getting all of the information and the data to be able to bring back to formulate what they needed moving forward."*
	Reflective Questioning	*"They might need to hear more about this or that. And I think that's good, but I think we need, if you're talking about working in a*

	coaching relationship, you need a coach that asks a lot of questions like that, so that they know they don't really need to be focusing on, they need this, or you have to… I really feel like that's imperative. You've got to ask the questions so that you're relevant. So that those schools will be like, 'okay, yeah, we're listening because we hear what you're saying, and we need this.'"
Data Support	*"Specifically, the first thing is just helping us to analyze the data. They actually did that task for us. And helping us create awareness as to where we have to go next."*
Needs and Resource Assessment	*"It actually was the metrics, like the actual inventory. Giving that data and that information directly to the curriculum instruction and saying, 'this is what [District Leader] and I see, but it's not all-encompassing,' but literally gave them and sent it to them. And that's when they began to, over the summer, figuring out we are not using the right approach, we're not using the evidence-based strategy. I didn't realize that we actually have people that can help guide, and also there are templates, and we're making things up on our own. So that inventory literally, actually, by completing that last year, gave a thrust to say we did not prioritize behavioral mental health."*
Goal Setting	*"An assessment of where you are with [SBH], I think that's helpful to start off with. And then you set a goal, or three goals. That was really helpful because it helped me frame...'okay, these are my marching orders.'"*
Connection to Resources	*"And then realistic interventions, interventions, realistic skills, realistic tips, things that we can implement quickly. And then other things that take a little longer, right? We need some quick fix, and we need some things that we can slowly roll out throughout the year. I think they did a good job with that with us, too, as things that were instant that we could work on, and then things that we had to*

		bring back, or they had to bring back to us from those meetings and sessions to things that we could work on and roll out throughout the year."

Supplemental Table S4

Thematic Structure of Barriers and Facilitators- Organizational Factors

Theme	Subtheme	Exemplar Quotes
Infrastructure	Teams	*"I think that you have to have a district MTSS team. I really believe that. It can be kind of informal, it doesn't have to be fully enhanced, doesn't have to be every single stakeholder. It doesn't have to have all the pieces that we know that are evidence-based and researched. But, having some district-level teaming that is willing to meet on a regular basis, be it monthly or quarterly. I wouldn't recommend quarterly, but at least monthly, that would be great."*
	Time	*"Right now I feel like the barrier is the limited amount of time when you're able to pull everyone together. All we have is the PD days at the beginning of the school year, and it's typically one to two days, and with the one to two days, there's some PD trainings that are mandatory based upon your area of what you do within the district. So, being able to have our SBHA coaches be able to literally touch more people during training is very limited, because we don't have the amount of time built in to be able to have her speak to more groups."*
	Staffing	*"There's no way around having at least a school-based therapist or social worker in every school dedicated to working towards students'*

	mental health needs. I feel like that has to be in place. Of course, every school has a school counselor, but every school doesn't have a therapist or a social worker that's actually dedicated to that one school...a lot of districts utilize one or two people districtwide, so that lends towards having a mediocre level of services because you're pulled in too many different directions for it to be successful."
Financial Resources	*"Having the coach come on board, I feel like helped impact my team be able to have a budget to be able to go to more trainings, to be able to implement, to have the resources to buy more therapeutic resources to utilize in their offices. My team hasn't had a real budget ever. We got our first account number, maybe six or seven years ago, there was an account, but it only covered ink and occasionally a couple of board games. Now, our coach helped put that in place, so every therapist got $500 worth of resources that they can utilize in their offices and be able to help the students and they're so excited."*
Community Resources	*"One thing that I've been saying for a long time is that there are a lot of resources for kids beyond the elementary age, but there's not a lot of resources in the community for elementary age children, like respite type of situations or with things that would help the parents that doesn't require an extended waiting period—besides going to mental health in a crisis, but we don't want them to always be in a crisis situation before they're getting the help that they need."*
Leadership Support	*"We've been very blessed with a superintendent that is 100% supportive of mental health...He's just phenomenal, and he's really empowered us to do what we need to do, and to support us when a parent's upset with us because we've had to send their child to the hospital. He's the one that's like, 'okay, tell me the steps you took. Tell me what you did. Did y'all follow our protocols?' We followed our protocols, we took our steps. He's like, 'then you're good. We did the right thing for kids, and we're always going to do the right things for kids.'"*

	Access to Data	*"To be able to actually keep good coaches, you've got to have good data."*
Norms	Collaboration	*"You see some of that or this interplay of people trying to, if in a meeting, trying to share information to one-up each other. And it's not a collaboration. It's literally, 'I'm going to share this because this is going to make me look good.' And the next person comes in at this angle. And then leadership almost encourages that. That can be very difficult."*
	Hierarchical Relationships	*"Egos. If they truly believe that they are the expert, that it's their school, you have that kind of culture within and you feel it. You can walk into a school and even before anybody says anything, it's cold. Even though it could look beautiful, you feel a coldness...I think that an be a tremendous barrier to being able to actually have a partnership."*
	Family/Community Partnerships	*"I think that if you have schools that are in a community that does not empower the community voice or the parent voice or the student voice, then that does create more rigidity. It is a top-down approach versus actually listening to those that are doing the actual work. I think that could be a very big challenge, as well."*
	Accountability	*"Well, one thing is for certain, even though the teams were having the support team meeting, sometimes the importance of everyone showing up wasn't always conveyed until more so the accountability piece was there. The meeting can't flow without it. Make sure they're being present. You need everyone present to get feedback on what's happening."*
	Trust	*"I think making sure that the leader, the school, the staff, they're in a place where they can trust each other to be vulnerable. They have*

		some honest conversations, because the relationships in my building are varying levels."
	Default Practices	*"A child is struggling in school, in the classroom, and immediately calling for administration is something that does happen often."*
Mindsets	Need to change	*"Let's say when you get sick, you're like, 'okay, I want to go to the doctor, I want to learn about this, I want to get this medicine, because I am personally feeling this ailment.' Okay? So, I feel like that has to be in place, where you're like, 'okay, we need to work on this. There is this need. What can we do to address it? Where can we go to get help?' I feel like that need needs to be in place, where if it's not bothering you before, then you may have been like, 'it's not a problem, I'm not really worried about it,' and you don't really pursue trying to fix it or get better at something...You've got to realize there's this sense of urgency, and we need to address this."*
	Openness to change	*"I think that it also takes an openness for others to really come on board. And I think what's happening."*
	Capability to change	*"So I'm not necessarily saying that you're willing, that wouldn't be the biggest part of it, but certainly a part of it, but more so when I say ready, I mean...that there's an opportunity for alignment with what I'm providing. So there's an opportunity, there's a need, but then there's something in your personality, or something in your demeanor, or processes, or system that says, 'I'm capable…'"*
	Investment in change	*"It's waking up every morning, and you have leadership that says, 'I want, not just academic rigor, but to make this school truly feel safe. Not just physically, but actually emotionally. I want to teach kids empathy. I want kids to be able to do things in collaboration. I am wanting kids to be able to feel like if there's going to be a crisis, that they have strong adults in this building that are going to be able to support and serve.' That has to come from leadership."*

Shared Commitment	*"And we tell the teachers, 'we're all learning and working together. You can't get this wrong. There's no right or wrong way to do this, but let's just walk hand in hand.' Then we're going to get this done."*
